# A simple method for poly-D-lysine coating to enhance adhesion and maturation of primary cortical neuron cultures *in vitro*

**DOI:** 10.3389/fncel.2023.1212097

**Published:** 2023-06-21

**Authors:** Aurélie Stil, Benoît Liberelle, Dainelys Guadarrama Bello, Lucile Lacomme, Laurie Arpin, Pascale Parent, Antonio Nanci, Éric C. Dumont, Tarek Ould-Bachir, Matthieu P. Vanni, Gregory De Crescenzo, Jean-François Bouchard

**Affiliations:** ^1^École d’optométrie, Université de Montréal, Montreal, QC, Canada; ^2^Département de Génie Chimique, Polytechnique Montréal, Montreal, QC, Canada; ^3^Faculté de Médecine Dentaire, Université de Montréal, Montreal, QC, Canada; ^4^Département de Biochimie et Médecine Moléculaire, Faculté de Médecine Dentaire, Université de Montréal, Montreal, QC, Canada; ^5^Department of Biomedical and Molecular Sciences, Queen’s University, Kingston, ON, Canada; ^6^Département de Génie Informatique et Génie Logiciel, Polytechnique Montréal, Montreal, QC, Canada

**Keywords:** primary cortical neuron cultures, glass coverslip, poly-D-lysine grafting, neuronal adhesion, neuronal maturation, synaptic contacts, synaptic activity, neuronal networks

## Abstract

**Introduction:**

Glass coverslips are used as a substrate since Harrison’s initial nerve cell culture experiments in 1910. In 1974, the first study of brain cells seeded onto polylysine (PL) coated substrate was published. Usually, neurons adhere quickly to PL coating. However, maintaining cortical neurons in culture on PL coating for a prolonged time is challenging.

**Methods:**

A collaborative study between chemical engineers and neurobiologists was conducted to find a simple method to enhance neuronal maturation on poly-D-lysine (PDL). In this work, a simple protocol to coat PDL efficiently on coverslips is presented, characterized, and compared to a conventional adsorption method. We studied the adhesion and maturation of primary cortical neurons with various morphological and functional approaches, including phase contrast microscopy, immunocytochemistry, scanning electron microscopy, patch clamp recordings, and calcium imaging.

**Results:**

We observed that several parameters of neuronal maturation are influenced by the substrate: neurons develop more dense and extended networks and synaptic activity is enhanced, when seeded on covalently bound PDL compared to adsorbed PDL.

**Discussion:**

Hence, we established reproducible and optimal conditions enhancing maturation of primary cortical neurons *in vitro*. Our method allows higher reliability and yield of results and could also be profitable for laboratories using PL with other cell types.

## 1. Introduction

In 2012, Millet and Gillette published a historical review on origins and progress of neuron culture methods (Millet and Gillette, 2012). As illustrated on Figure 2A of their manuscript, glass coverslips are used as a substrate since the very first experiments with nerve cell cultures, conducted by Harrison in 1910 (Harrison, 1910). The main advantage of glass substrate is to allow the observation of cells, using contemporary techniques, including optical, electron, fluorescent, or confocal microscopy.

Plastic has been used later as substrate for cell cultures. Glass and plastic surfaces were first used without any coating, but a high percentage of neurons failed to adhere and were eliminated at the first change of medium (Yavin and Menkes, 1973). In 1974, the very first study of brain cells seeded onto polylysine (PL) coated substrate was published (Yavin and Yavin, 1974). This study was based upon previous observations, published almost 20 years before, on the electrostatic interactions between the positively charged lysine groups of PL and the negative phospholipid bilayer of the plasma membrane (Nevo et al., 1955).

PL is still being used for neural cultures as seen in the handbook cited hereafter (Doering, 2010). It includes 23 methodological articles for neural cultures, constituting a sample and a non-exhaustive report of the popularity of this substrate. However, this handbook is an example of choice showing that no consensus exists (1) on the recipe to prepare the PL solution, and (2) the coating procedure of substrates. Indeed, PL (existing as two enantiomers, namely poly-D-lysine (PDL) or poly-L-lysine (PLL) is dissolved in different solutions including ultra-pure water, phosphate (PBS) or borate buffers, and is used alone or in combination with other coating molecule (e.g., laminin). Final concentrations of PL range between 10 μg/ml and 1 mg/ml, and incubation with plastic or glass substrate is performed at various temperatures (room temperature (RT) or 37°C) for minutes to hours. PL-covered surfaces are rinsed with water, or PBS, and then left to dry, or the surface is immediately covered by plating medium. Each laboratory also has its own way to clean the glass surface, using sterile distilled water, ethanol, xylene, or nitric acid (Doering, 2010).

Usually, neurons rapidly adhere on PL coating. However, maintaining neurons in culture on PL coating for a long period of time (i.e., more than 7 days) is a challenge. As early as in 1986, a team from the Department of Biology, at the Texas Woman’s University, published intriguing results about the evolution of spinal cells growing on PL. Although showing a good adherence and a typical morphological differentiation during the first period after seeding, cells were seen to re-aggregate after two or three weeks in culture, and look like cells seeded without polylysine (Lucas et al., 1986; see Figure 4B).

In our laboratory, we are used to relying on PDL because it is inexpensive, known as a well-characterized universal cell substrate, not degraded by cells, and, because it is a synthetic polymer, PDL is stable in solution. PDL is dissolved in sterile ultra-pure water (20 μg/ml at pH 6; named PDL6) and adsorbed on coverslips. Neuronal adhesion occurs quickly and efficiently with this method. However, we observe a high variability between our primary cortical neuron cultures, and most of the time, long-term maturation is compromised. Interestingly, our cultures follow a similar evolution to that described in 1986 (Lucas et al., 1986). Indeed, neurons are usually seen to reaggregate, randomly one week or later after seeding, depending on each culture.

As seen in the literature, some laboratories have already tried to improve cell adhesion and several strategies were previously employed to chemically graft PDL on glass substrate. We here report the development of a simple and effective substrate, promoting both adhesion and maturation of neuronal cells. In this work, the simplest and the safest way to generate covalently grafted PDL was used. More specifically, a one-step grafting approach involving the epoxy moieties of (3-glycidyloxypropyl)trimethoxys (GOPS) was chosen, as no toxic reagent such as glutaraldehyde is required. Moreover, for the sake of convenience, GOPS was deposited in gas phase at RT, avoiding the use of organic solvents.

The presence of the cationic PDL was qualitatively evaluated using an anionic dye, namely Coomassie Brilliant Blue (CBB) (Noel et al., 2011). This rapid and economical colorimetric method was performed to easily characterize the PDL layer. Using the CBB method, we compared the standard adsorption method and our method of PDL covalent binding onto glass coverslips. We have also investigated the impact of polymer deposition parameters (pH, concentration, time) on the density, homogeneity, and reproducibility of the PDL layer.

We next studied adhesion and maturation of primary cortical neurons with many complementary morphological and functional approaches including phase contrast microscopy, immunocytochemistry (ICC), scanning electron microscopy (SEM), patch clamp recordings and calcium imaging. We observed that several parameters of synaptic maturation were influenced by the substrate. Indeed, neurons seeded on grafted PDL dissolved at pH 9.7 (named GPDL9) developed more dense and extended networks, and synaptic activity was enhanced, compared to neurons seeded on adsorbed PDL6. Hence, we established here reproducible and optimal conditions enhancing a healthy maturation of primary cortical neurons *in vitro*.

## 2. Materials and equipment

### 2.1. Reagents

Gibco*™* products (Hank’s Balanced Salt Solution (HBSS), Neurobasal*™* medium, Minimum Essential Media Suspension (S-MEM), B-27*™*, GlutaMAX*™* supplement, penicillin-streptomycin and trypsin), and secondary antibodies (Alexa) Fluor*™* 488 Goat anti Rabbit (#A11008), Alexa Fluor*™* 546 Goat anti Mouse (#A11003) were purchased from Life Technologies (Burlington, ON, Canada). Poly-D-Lysine (PDL) powder, DNAse, HCl, (3-glycidyloxypropyl)trimethoxysilane (GOPS), MgCl_2_, K-Gluconate, Na_2_CO_3_, K_2_CO_3_, ATP, GTP, Ethanolamine (EtA), Coomassie Brilliant Blue (CBB), primary antibody against synaptophysin (#S5768, RRID:AB_477523) and Hoechst 33258 were purchased from Sigma-Aldrich (Oakville, ON, Canada). Primary antibody directed against the GluR1 subunit of the AMPA glutamate receptor (#AB1504, RRID:AB_2113602) was obtained from Millipore (Oakville, ON, Canada). Bovine serum albumin (BSA) was purchased from Hyclone (Logan, UT, USA), and trypan blue from MP Biomedicals (Irvine, CA, USA). Normal goat serum (NGS) was purchased from Jackson ImmunoResearch (West Grove, PA, USA). Viral vectors were purchased from the Canadian Neurophotonics Platform Viral Vector Core Facility (RRID:SCR_016477; Quebec, QC, Canada). The coverslip mounting medium Immu-Mount*™*, Tween 20, NaOH, Paraformaldehyde (PFA), HEPES, NaHCO_3_, NaCl, MgSO_4_, dextrose, KOH and isopropanol were obtained from Fisher Scientific (Edmonton, AB, Canada), KCl, KH_2_PO_4_ and EGTA from JT Baker (Philipsburg, NJ, USA), CaCl_2_ from EMD Chemicals (Gibbstown, NJ, USA), tetrodotoxin (TTX) from Tocris (Toronto, ON, Canada), and picrotoxin (PTX) from Abcam Biochemicals (Toronto, ON, Canada). Alternum, an ethical substitute for Fetal Bovine Serum (FBS) was purchased from Alterna Technologies (Stoke, QC, Canada), glutaraldehyde and osmium tetroxide from Electron Microscopy Sciences (EMS; Hatfield, PA, USA).

### 2.2. Preparation of poly-D-lysine solutions

Poly-D-lysine (PDL; molecular weight of 70–150 kDa) is prepared in sterile ultra-pure water, dissolving 10 mg in 250 ml for a final concentration of 40 μg/ml. The pH of this solution is measured to be 6, and this solution was named PDL6. It is diluted with ultra pure water to obtain PDL solutions at 1, 5, 10, and 20 μg/ml. PDL9 solutions are obtained using PDL6 solutions (1, 5, 10, 20, and 40 μg/ml), by adding sodium carbonate (final concentration of 50 mM), and then adjusting pH at 9.7 with 1M HCl. All solutions are sterilized through a 0.2 μm filter.

### 2.3. Equipment

CKX41 phase contrast microscope and FV300 confocal microscope (Evident Canada, Quebec, QC, Canada), Critical Point Dryer (Leica EM CDP3000; Leica Microsystems Inc., Ontario, Canada), ultra high-resolution scanning electron microscope (SEM) Regulus 8220 (Hitachi, Ltd., Tokyo, Japan), Digidata 1322A interface, Axopatch 200B amplifier and pCLAMP software (Axon Instruments^®^, Molecular Devices, San Jose, CA, USA), Image Pro Plus software (Media Cybernetics Inc., Rockville, MD, USA), Prism software (Graph Pad Software, San Diego, CA, USA), Matlab (The MathWorks, Inc., Natick, MA, USA).

## 3. Methods

### 3.1. Preparation of PDL coated coverslips

#### 3.1.1. PDL adsorption

PDL6 or PDL9 is deposited as a drop of 200 μl onto glass coverslips, during 10, 20, 30, or 100 min. To rinse PDL, coverslips are dipped in a 50 ml tube containing sterile ultra-pure water. PDL-coated coverslips are then placed in 24-well plates and covered with sterile ultra-pure water until neuron seeding (occurring on the same day).

#### 3.1.2. PDL chemical grafting strategy

**FIGURE 1:**
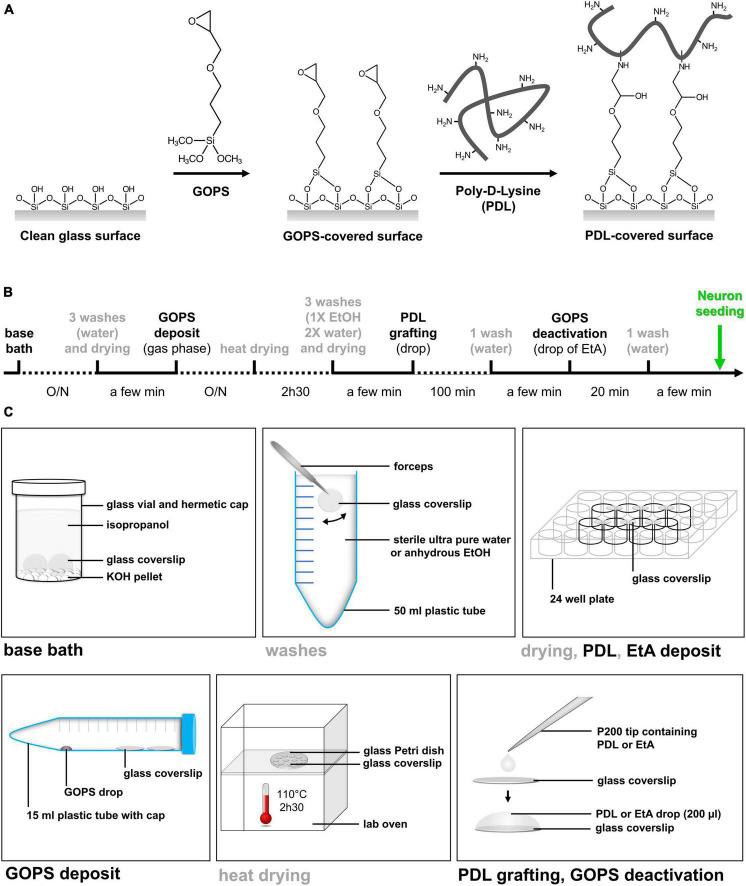
Simple method of PDL9 covalent binding on glass coverslip. **(A)** Schematic illustration of the chemical grafting procedures. The grafting of GOPS on glass surfaces is followed by chemical immobilization of PDL chains. **(B)** Timeline and duration of the different steps from bath base coverslip cleaning to neuron seeding. **(C)** All steps (base bath, washes, drying, silane deposit, heat drying, PDL grafting and EtA deposit) are illustrated.

To stabilize the PDL layer on the coverslip surface, we developed an easy and reproducible method for the covalent grafting of PDL onto the glass substrate (Figure 1A).

The first step consists in transforming poorly reactive silanol groups of glass substrate into chemically active functionalities. Among them, amino or epoxy groups were usually preferred and generated via a silanization procedure involving aminopropyltriethoxysilane (APTES) or (3-glycidyloxypropyl)trimethoxysilane (GOPS), respectively (Bordoni et al., 2002; Wu et al., 2005; Kim et al., 2011; Xing et al., 2012; Kamande et al., 2019). Both silane layers can be deposited on glass surfaces, in gas phase or in organic solution. A short curing step in an oven was then required to chemically attach silane molecules to glass substrate (Smith and Chen, 2008). Finally, APTES- and GOPS-exposing surfaces were reacting with the primary amino groups distributed along the PL chains. For APTES-coated surfaces, bifunctional linkers such as glutaraldehyde or bissulfosuccinimidyl suberate are required (Kim et al., 2011; Kamande et al., 2019). For GOPS-coated surfaces, no additional linker was needed to graft PL.

More specifically, GOPS is first deposited here on clean glass coverslips in gas phase at room temperature. GOPS-deposited layer is then heated to promote condensation reactions with the glass surface leading to chemically grafted GOPS. Glycidyloxy rings are finally reacted with the deprotonated amino groups of the PDL chains (pKa 9.5) (Franzin and Macdonald, 2001). Note that PDL is dissolved in a carbonate solution (pH 9.7, named PDL9), ensuring that amino groups distributed along the chain are mostly deprotonated.

#### 3.1.3. PDL covalent binding: detailed protocol

The proposed PDL grafting method is easy to perform in a conventional wet laboratory and does not require specific material. The entire sequence of manipulations allowing covalent grafting of PDL at pH 9.7 (PDL9) on glass coverslips, takes about 48 h (Figure 1B). Every step is detailed in Figure 1C, and Table 1 lists all lab consumables, tools and reagents that are required at each step of our protocol, to coat a batch of 24 coverslips.

**TABLE 1:** Material required at each step, wash, drying or for storage to coat a batch of 24 coverslips.

Main steps	Materials	Washes, drying or storage	Materials
Base bath	6 glass vials 24 glass coverslips Isopropanol KOH pellets Forceps pair #1	3 washes and drying	Sterile ultra pure water 3 × 50 ml plastic tubes Forceps pair #1 24-well plate #1
GOPS deposit	GOPS 1 ml syringe 22G needle 12 × 15 ml plastic tubes Lab oven (set at 110°C) Glass Petri dish and lid Forceps pair #2	3 washes and drying	Anhydrous EtOH Sterile ultra pure water 3 × 50 ml plastic tubes Forceps pair #2 24-well plate #2
PDL grafting	PDL pH 9.7 in carbonate buffer P200 and tips 24-well plate #2	1 wash	Sterile ultra pure water 1 × 50 ml plastic tubes Forceps pair #2 24-well plate #3
GOPS deactivation	EtA (1M, pH 9.15) P200 and tips	1 wash	Sterile ultra pure water 1 × 50 ml plastic tubes Forceps pair #2 24-well plate #3
		Storage until seeding	Sterile ultra pure water 24-well plate #4

That forceps #1 are only used for dipping in bath base and washes, and forceps #2 for all other dipping steps.

Coverslips are first cleaned overnight in vials containing a base bath (KOH pellets submerged with isopropanol; each vial containing 4 coverslips, held vertically), then washed with sterile ultra-pure water (surface of coverslips is hydrophilic) and let to dry for a few minutes (the surface of coverslips should be completely cleaned and free of any trace). Note that the base bath is originally transparent, but will become yellow to brown over time, while still being efficient. However, it could be cleaned with ultra-pure water and renewed when most of the KOH pellets have melted.

GOPS is deposited as a drop with a syringe at the bottom of 15 ml tubes. Grafting of GOPS is performed by placing the coverslips in these tubes (up to 4 coverslips per tube). Vapors of GOPS are let to adsorb on glass substrates overnight at room temperature (RT). Coverslips are then placed in a glass Petri dish and heated in a lab oven for 2h30 at 110°C. The surfaces are rinsed by soaking them in anhydrous ethanol (one time) and in sterile ultra-pure water (two times) and dried. Surface of coverslips should be homogeneously hydrophobic—if not, coverslips should be discarded. At this point, coverslips can be stored for many weeks in clean Petri dishes, protected from dust and light.

Otherwise, PDL9, i.e., PDL solution prepared in sodium carbonate buffer (pH adjusted at 9.7 using 1 M HCl), is deposited as a drop onto coverslips for 10, 20, 30, or 100 min, and then washed with sterile ultra-pure water. The unreacted GOPS sites were deactivated by covering the surfaces with ethanolamine (1M EtA in ultra-pure water, pH adjusted to 9.15 using 5M NaOH) for 20 min. Excess EtA was washed with sterile ultra-pure water. The PDL-grafted coverslips were placed in 24-well plates and covered with sterile ultra-pure water until neuron seeding (occurring on the same day).

Note that coverslips cleaning and GOPS deposit are performed under a chemical fume hood, and washes of coverslips after heating and deposit of PDL and EtA are performed under a laminar flow hood.

### 3.2. Colorimetric assay on glass surfaces

In previous works, the presence of the grafted PDL layer was confirmed using contact angle measurements (CAM) or X-ray photoelectron spectroscopy (XPS) (Kim et al., 2011; Xing et al., 2012). In order to precisely localize the PL layer on the glass surface, PDL chains tagged with fluorescent molecules were also exploited (Kim et al., 2011). Finally, atomic force microscopy (AFM) was used to characterize the homogeneity of the PDL layer (Xing et al., 2012).

Here, PDL density and homogeneity on glass coverslips were qualitatively characterized using a colorimetric method, i.e., Coomassie brilliant blue method, based on a previously reported work (Noel et al., 2011). Briefly, PDL-coated coverslips were incubated in a CBB solution (0.5 mg/ml in acidic solution pH 2.2; 85:10:5 v/v ultra-pure water/methanol/acetic acid) for 5 min at RT, rinsed by dipping the surfaces in the acidic solution to remove unbound dye and dried. Coverslips were then immersed in 250 μl of an alkaline desorption solution (pH 11.25; 0.125 M K_2_CO_3_ in 50:50 v/v ultra-pure water/methanol). The pH of the solution containing the desorbed dye was adjusted to pH 3 by adding 10% of 3 M HCl. The absorbance of the solution containing the desorbed CBB was measured at 620 nm.

### 3.3. Animals

All procedures were performed in accordance with the guidelines from the Canadian Council on Animal Care for the care and use of laboratory animals and were approved by the ethics committee on animal research of the *Université de Montréal* (*CDEA*; protocol numbers #21-058, #21-059, #22-057 and #22-058). C57bl/6J wild-type (WT) mice were purchased from Jackson Laboratory and maintained in an environmentally controlled room held at 22°C, with 12 h dark/light cycle. Food and water were provided *ad libitum*.

### 3.4. Cultures of primary cortical neurons

Embryos (E15.5-E17) were surgically obtained from gestating mice. Brains were dissected in HBSS. Cortices were digested in 2 ml S-MEM containing 2.5% trypsin and 2 mg/ml DNase for 15 min at 37°C. The pellet was transferred into 10 ml S-MEM with 10% Alternum (a substitute for Foetal Bovine Serum (FBS)), on ice. The precipitate was then transferred into 2 ml S-MEM with 10% Alternum for mechanical dissociation. The supernatant was transferred in 5 ml Neurobasal medium supplemented with 2% B27, 100 U/ml penicillin, 100 μg/ml streptomycin and 0.5 mM of GlutaMAX*™*, and the cell suspension was passed through a strainer (40 μm pore size). Dissociated cells were counted and plated at 40,000 per well for cell adhesion observation (at Day *in vitro* 4 (DIV4)), and 75,000 cells per well for all others experiments.

### 3.5. Phase contrast microscopy

Cultures seeded at 40,000 cells per well, were imaged at DIV4, through 24-well plates, with a CKX41 phase contrast microscope (Evident Canada, Quebec, QC, Canada). Depending on PDL conditions, cells were observed as the three following main types: non-adherent small clusters of brilliant cells, individual gray adherent neurons with various shapes and developing neurites, or individual small round brilliant cells.

### 3.6. Immunocytochemistry and confocal microscopy

Primary cortical neuron cultures were washed with PBS (0.1 M, pH 7.4), fixed 15 min in 4% PFA, washed again with PBS and blocked with 2% NGS and 2% BSA in PBS containing 0.1% Tween 20 for 30 min at RT. Neurons were then incubated in the blocking solution containing anti-synaptophysin (1:500) and anti-GluR1 (1:500) antibodies, overnight at 4°C. The following day, wells were washed, and cultures were labeled with Alexa Fluor*™* secondary antibodies (488 and/or 546). Nuclei were stained with Hoechst 33258. The coverslips were mounted on slides using a commercial mounting medium (Immu-Mount) and imaged with a FV300 confocal microscope (Evident Canada, Quebec, QC, Canada). For whole coverslip mapping, about 100 images were automatically acquired at 8 × magnification, and then stitched using the Fluoview software. Neuronal networks and individual neurons were acquired respectively at 20× and 100× magnification.

### 3.7. Analysis of pixel density

Synaptophysin pixel density was quantified from the 8 × maps with the Fiji software using image adjusted threshold, measure and particle analyse tools. Pixel density was expressed as a% of the total analyzed area corresponding to the whole coverslip. For cluster quantification, data are expressed as a% of PDL6 20 μg/ml.

### 3.8. Analysis of synaptic contacts

Synaptic contacts were automatically detected using the Intellicount an open-source code/software (Fantuzzo et al., 2017), modified to be compatible with Microsoft Windows operating system. Some modifications were also done to detect only the soma of the main neuron in the field. GluR1 positive neurites and soma were detected and delimited respectively with green and blue edges, and red positive punctae of synaptophysin were identified and delimited as red dots. Extracted data were neurite and soma area, as well as density and size of synaptic punctae. Data were then expressed as a% of PDL6.

### 3.9. Scanning electron microscopy

DIV10 and DIV14 primary cortical neurons were washed with PBS (0.1 M, pH 7.4), fixed 1h in 2.5% glutaraldehyde at 4°C, washed again with PBS, post-fixed for 1h with 1% osmium tetroxide at 4°C, then washed with ultra-pure H_2_O. Cells were dehydrated through an ethanol gradient (30, 50, 70, 80, 90, 95, and 100% twice) and dried in a Critical Point Dryer (Leica EM CDP3000; Leica Microsystems Inc., Ontario, Canada). After carbon coating, cultures were imaged with an ultra high-resolution scanning electron microscope (SEM) Regulus 8220 (Hitachi, Ltd., Tokyo, Japan) operated at 1 kV.

### 3.10. Electrophysiology

Electrophysiological recordings were performed on neurons perfused with oxygenated (95% O_2_, 5% CO_2_) artificial Cerebro-Spinal Fluid (aCSF; in mM: 124 NaCl, 3 KCl, 1.25 KH_2_PO_4_, 1.3 MgSO_4_, 1.6 CaCl_2_, 26 NaHCO_3_, and 10 dextrose), at RT. Patch pipettes (4–6 MΩ) were filled with a solution containing (in mM) 140 K-gluconate, 5 NaCl, 2 MgCl_2_, 10 HEPES, 0.5 EGTA, 2 ATP, and 0.4 GTP (pH 7.2–7.3; osmolarity 275–285 mOsm). Whole cell recordings were performed through a Digidata 1322A interface (Axon Instruments^®^, Molecular Devices, San Jose, CA, USA). Voltages were corrected off-line for the liquid junction potential (15 mV). Neuron properties were recorded in current clamp mode at -80 mV. Spontaneous miniature excitatory postsynaptic currents (mEPSCs) were recorded in voltage-clamp mode at -70 mV in the presence of 1 μm TTX and 50 μm picrotoxin. Data were sampled at 10 kHz and filtered at 10 (current clamp mode) and 1 kHz (voltage clamp mode), using an Axopatch 200B amplifier. Acquisition and analysis were performed with pCLAMP (Axon Instruments^®^, Molecular Devices, San Jose, CA, USA).

The resting membrane potential was read 1–2 min after patching, in current clamp mode without any current injection (I = 0). Then, the protocol to generate spikes consisted of injecting 1 s depolarizing current pulses of 5 pA increments at a holding potential of -80 mV. The rheobase was the minimum current required to elicit an action potential.

Hyperpolarizing and depolarizing current pulses of 1 s and 30 pA increments were injected. Membrane potentials were plotted against injected currents to get voltage-current (V-I) relationship. The input resistance was calculated as the slope of a straight line fitted to the V-I curve.

A train of spikes was generated by suprathreshold current injection equivalent to twice the rheobase. The number of spikes evoked by a 1 s current pulse (mean of 3 repeated measures) was defined as the mean firing frequency.

The protocol to analyse action potential (AP) parameters, consisted of injecting 500 ms depolarizing current pulses of 5 pA increments. We analyzed parameters of the first elicited AP. AP threshold potential was defined as the voltage where the rising slope reached 10 mV/ms. Duration was the time to reach the half-maximum peak from the threshold. Amplitude was the voltage difference between the half-maximum peak and the threshold.

Then, single spikes were elicited with 10 ms current pulses of 5 pA increments. The peak amplitude and duration of the fast afterhyperpolarization (AHP) were measured from the threshold potential.

### 3.11. Calcium imaging

Cultures were infected at DIV2 with the following viral vector: AAV2/php.eB-hSyn-GCaMP6s (titer: 1.5E13 Genome Copies/ml; diluted at 1/1000 in culture medium). Green fluorescence was detected with a FITC filter set. Spontaneous changes of fluorescence were recorded at DIV9 with the Image Pro Plus software (Media Cybernetics Inc., Rockville, MD, USA). Images (1600 × 1200 pixels) of the calcium spontaneous fluctuation were extracted and analyzed using Matlab (The MathWorks, Inc., Natick, MA, USA). The sequences of ΔF/F activity were computed for each pixel as the difference between the value of each frame subtracted and divided by the minimum value of the whole sequence. The maximum projections of ΔF/F over time were then calculated and thresholded by a binary mask of the average neuronal fluorescence higher than 1σ of distribution of the whole image. The resulting image (Maximum ΔF/F) was then used to identify the pixels showing the highest fluctuations. Curves of ΔF/F (%) were represented graphically over time for 4 neurons in each condition.

### 3.12. Statistics

Data were expressed as the mean ± standard deviation (SD), or as a% ± SD of a given group, as specified, and were represented and analyzed through the Prism software (Graph Pad Software, San Diego, CA, USA). Although being independent measures, some data were presented as curves of connected dots to improve result readability. Data normality was determined with the D’Agostino-Pearson omnibus test. Statistical significance of differences between groups was evaluated by unpaired t-test, Mann and Whitney test, Kruskal-Wallis test (and Dunn’s post-test), two-way ANOVA or mixed effect model (and Tukey post-test), or Kolmogorov-Smirnov test, depending on the experimental design.

## 4. Results

### 4.1. The extracellular matrix of adsorbed poly-D-lysine is responsible for neuron reaggregation, after a few days in culture

**FIGURE 2:**
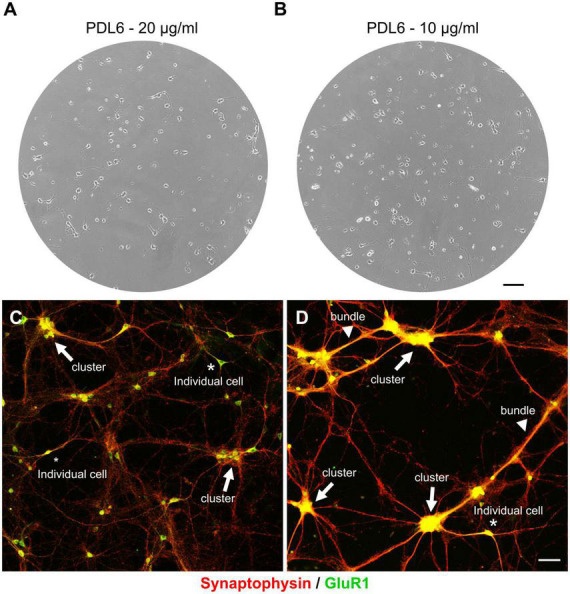
The extracellular matrix of PDL6 is responsible for neuron reaggregation, after a few days in culture. Phase contrast images of DIV4 cultures. Neurons showed a good adhesion when seeded on adsorbed PDL6 (20 μg/ml **(A)** and 10 μg/ml **(B)**. **(C,D)** DIV10 culture were immunostained for synaptophysin in red, and GluR1 in green. Neurons seeded on PDL6 20 μg/ml **(C)**, tended to reaggregate into clusters (arrows), and only a few individual glutamatergic neurons were visible (asterisks; GluR1 positive green cells). With a lower concentration of PDL6 [10 μg/ml **(D)**], reaggregation was more important, clusters were connected with thick bundles (arrowheads) and individual neurons were very rare **(A,B)**: scale bar = 100 μm; **(C,D)**: scale bar = 50 μm.

We are interested to find pathways involved in axonal guidance or growth (Argaw et al., 2011; Duff et al., 2013; Cherif et al., 2015; Cherif et al., 2018; Laroche et al., 2021), and synaptogenesis (Goldman et al., 2013), using cortical neuron cultures from mouse embryos as model. We use antibodies against synaptophysin as a presynaptic vesicle marker (Jahn et al., 1985), and against GluR1, an AMPA receptor subunit, as a postsynaptic marker (Hollmann et al., 1989). Studying synaptic contacts requires healthy neurons for 10 days *in vitro* (DIV) and beyond. Unfortunately, we have observed a high variability between our cultures, and only a few could be used to investigate synaptogenesis. After many unsuccessful changes and tests in our protocol, we hypothesized that variability could come from the extracellular matrix of adsorbed poly-D-lysine (PDL). PDL was prepared in ultra-pure water at 20 μg/ml (pH 6; named PDL6) to coat glass coverslips. Although showing a good adhesion the first days after plating (Figures 2A, B), neurons seeded on PDL6 (20 μg/ml; Figure 2C; synaptophysin, in red and GluR1 in green, respectively as pre- and postsynaptic markers), tended to reaggregate into clusters (indicated by arrows). It occurred randomly and slyly, a few days after seeding, the seventh day or after. Figure 2C is an example of clustering seen at DIV10, and only a few individual glutamatergic neurons are visible (asterisks; GluR1 positive green cells). Interestingly, with a lower concentration of PDL6 (10 μg/ml; Figure 2D), reaggregation was more severe, clusters were connected with thick bundles (arrowheads) and individual cells were rare. At 10 μg/ml, clusters were significantly increased by 63 ± 15.7% compared to 20 μg/ml (t-test, ***p < 0.001; *n* = 3 cultures per condition). As mentioned by Lucas et al. in 1986 (Lucas et al., 1986), reaggregation may be explained by “a gradual change in the nature of cell adhesion and a concurrent increase in cell mobility”, and we hypothesized that our observations are due to the PDL6 layer becoming unstable with time.

### 4.2. PDL staining on glass coverslips and deposit characterization

**FIGURE 3:**
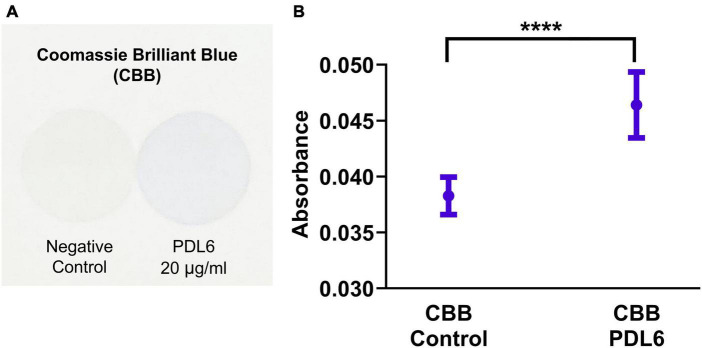
Detection of PDL layer on coverslips. **(A)** Images of coverslips stained with Coomassie Brilliant Blue (CBB) dye: glass surfaces without PDL coating (negative control, left) and coated with PDL6 at 20 μg/ml (right). **(B)** Absorbance values of the solutions containing desorbed CBB staining (unpaired t-test; ****p < 0.0001; *n* = 10–11 per condition).

In this work, we used the Coomassie Brilliant Blue (CBB) staining technique to characterize both the presence and the homogeneity of the PDL coatings on coverslips (Noel et al., 2011). This fast and easy colorimetric technique was found to successfully stain PDL6 (20 μg/ml) layer (Figure 3A). Absorbance measurements of the solutions containing desorbed CBB consistently gave higher values for PDL6-coated coverslips compared to negative control (pristine glass surface) (Figure 3B; 20 μg/ml; unpaired t-test; ****p < 0.0001; *n* = 10–11 per condition).

**FIGURE 4:**
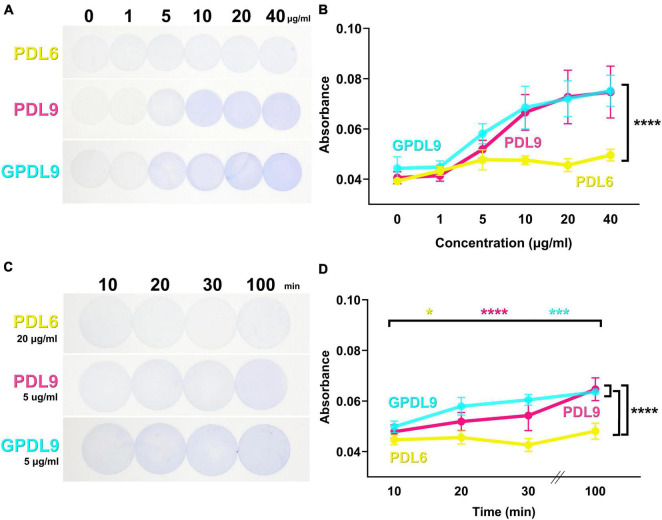
PDL6 and PDL9 staining for various concentrations and incubation times. **(A)** Coverslips coated with adsorbed PDL6 (upper panel), adsorbed PDL9 (middle panel), or grafted PDL9 (GPDL9; lower panel) for 20 min and stained with CBB dye. Concentration of PDL ranged from 0 to 40 μg/ml. **(B)** Absorbance measurements of CBB desorbed from coverslips. Values for PDL9 and GPDL9 were significantly different compared to PDL6 (two-way ANOVA and Tukey’s multiple comparisons test; ****p < 0.0001; *n* = 6–18 per group). **(C)** Coverslips coated with adsorbed PDL6 (20 μg/ml; upper panel), adsorbed PDL9 (5 μg/ml; middle panel), or grafted PDL9 (5 μg/ml; GPDL9; lower panel) and stained with CBB dye. Duration of PDL deposit ranged from 10 to 100 min. **(D)** Absorbance measurements of CBB desorbed from the coverslips. Significant differences between 10 and 100 min for the 3 tested conditions (Mixed effect model and Tukey’s multiple comparisons test PDL6: yellow; *p < 0.05; PDL9: magenta; ***p < 0.001; GPDL9: cyan; ****p < 0.0001; *n* = 6–21 per group) are indicated. The three curves were also significantly different from each other (two-way ANOVA and Tukey’s multiple comparisons test PDL6 and PDL9: ****p < 0.0001; PDL6 and GPDL9 ****p < 0.0001; PDL9 and GPDL9: ****p < 0.0001).

The CBB staining technique was then used to compare different PDL deposition conditions. More specifically, PDL chemical grafting was compared to PDL physical adsorption, while varying three deposition parameters, *i.e*., pH, PDL concentration and incubation time. First, coverslips were coated either with adsorbed PDL at pH 6 (PDL6; Figure 4A; upper panel), adsorbed PDL at pH 9.7 (PDL9; Figure 4A; middle panel), or with grafted PDL at pH 9.7 (GPDL9; Figure 4A; lower panel) for 20 min. Concentration of PDL was ranging from 0 to 40 μg/ml for all the three deposition conditions (Figures 4A, B). Figure 3A showed homogeneous staining of CBB on all coverslips, indicating that the PDL layers were deposited in a controlled manner. Figure 3B shows the variation of the absorbance values of desorbed CBB when varying the quantity of deposited PDL. Both adsorbed or grafted PDL9 induced a significant increase of CBB staining in a concentration dependent manner, compared to PDL6 (two-way ANOVA and Tukey’s multiple comparisons test; ****p < 0.0001; *n* = 6–18 per group).

We then set PDL6 and PDL9 concentrations at 20 and 5 μg/ml respectively and evaluated the influence of PDL incubation time on PDL surface density. Coverslips were coated with adsorbed PDL6 (Figure 4C, upper panel), adsorbed PDL9 (Figure 4C, middle panel), or grafted PDL9 (Figure 4C, lower panel), for 10, 20, 30, or 100 min, and stained with CBB dye. Interestingly, increasing PDL incubation time improved the PDL layer homogeneity between the edges and the center of the coverslip. Absorbance measurements of desorbed CBB showed significant differences between 10 and 100 min for the 3 tested conditions (Figure 4D; mixed effect model and Tukey’s multiple comparisons test; PDL6: yellow, *p < 0.05; PDL9: magenta, ***p < 0.001; GPDL9: cyan, ****p < 0.0001; *n* = 6–21 per group). The three curves were also significantly different from each other (Figure 4D; two-way ANOVA and Tukey’s multiple comparisons test PDL6 and PDL9: ****p < 0.0001; PDL6 and GPDL9 ****p < 0.0001; PDL9 and GPDL9: ****p < 0.0001). Incubation of 100 min is the best compromise to favor homogeneous PDL coating while avoiding that it dries, For the further experiments, PDL was then incubated for 100 min, as the homogeneity of the PDL layer was improved.

### 4.3. Influence of type, concentration, and deposit method of PDL upon cell adhesion

**FIGURE 5:**
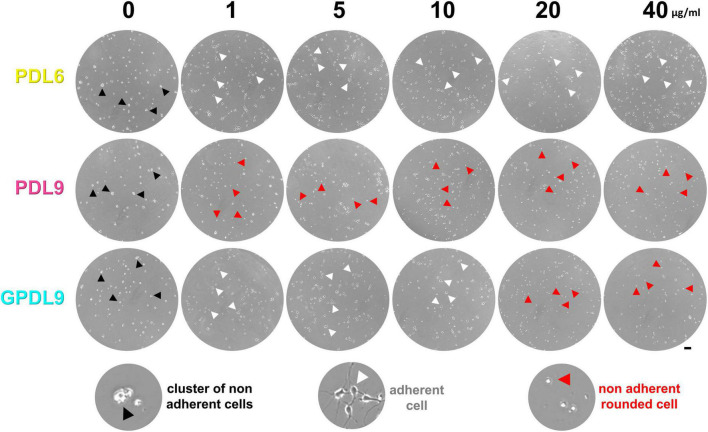
Early cell adhesion on various PDL substrates. Cultures seeded on PDL6 (upper lane), PDL9 (middle lane) or GPDL9 (lower lane), at 6 different concentrations (0, 1, 5, 10, 20, and 40 μg/ml), were imaged at DIV4 through the 24-well plates, with a phase contrast microscope. Depending on conditions, cells were observed as individual and gray adherent neurons with various shapes and developing neurites (white arrowheads), non-adherent small clusters of brilliant cells (black arrowheads), or individual small, rounded, and brilliant cells (red arrowheads; scale bar = 50 μm).

In the previous section, we highlighted major differences of CBB staining between our various conditions of PDL deposit. In the following parts, we investigated how cortical neuron cultures behaved on these different PDL substrates. Primary cortical neurons were seeded either on adsorbed PDL6 (Figure 5; upper panel), adsorbed PDL9 (Figure 5; middle panel), or grafted PDL9 (GPDL9; Figure 5; lower panel), at 6 different concentrations (0, 1, 5, 10, 20, and 40 μg/ml). Living cultures were imaged at DIV4 with a phase contrast microscope. When PDL coating was absent, cells did not adhere, and appeared as small clusters (Figure 5; black arrowheads). On PDL6 (1–40 μg/ml), and GPDL9 (1–10 μg/ml), cells were mostly seen as individual neurons with various shapes and developing neurites (Figure 5; white arrowheads). With adsorbed PDL9 (1–40 μg/ml) and high concentrations of GPDL9 (20 and 40 μg/ml), most cells did not adhere and appeared as small, brilliant, floating, rounded cells (Figure 5; red arrowheads).

### 4.4. Type, concentration, and deposit method of PDL highly influence neuronal network development

**FIGURE 6:**
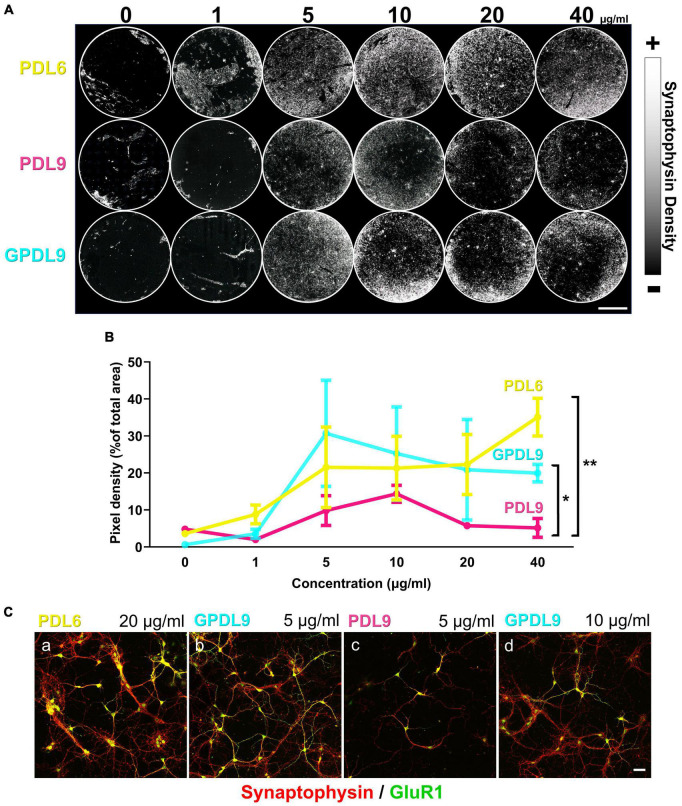
Influence of PDL type, concentration, and deposit method on neuronal network development. **(A)** Mapping of DIV10 neuronal cultures, grown on PDL6 (upper lane), PDL9 (middle lane) or GPDL9 (lower lane), at 6 different concentrations (0, 1, 5, 10, 20, and 40 μg/ml), immunostained for synaptophysin, a presynaptic vesicle protein. Synaptophysin density appeared as a gray scale (from black = 0 to white = 255; scale bar = 4 mm). **(B)** Quantification of pixel densities, expressed as a% of total area, obtained for each PDL condition: PDL6 (yellow curve), PDL9 (magenta curve) and GPDL9 (cyan curve). PDL9 is significantly different from GPDL9 and PDL6 (two-way ANOVA and Tukey’s multiple comparisons test; respectively, *p < 0.05 and **p < 0.01; *n* = 1–6 cultures per group). **(C)** Higher magnification (20×; synaptophysin in red and GluR1 positive cells in green) highlighted differences in the development of cultures, depending on PDL conditions (a: PDL6 20 μg/ml; b: GPDL9 5 μg/ml; c: PDL9 5 μg/ml; d: GPDL9 10 μg/ml;scale bar = 50 μm).

DIV10 cortical cultures, grown on PDL6 (Figure 6A; upper lane), PDL9 (Figure 6A; middle lane) or GPDL9 (Figure 6A; lower lane), at 6 different concentrations (0, 1, 5, 10, 20, and 40 μg/ml), were immunostained for synaptophysin, a vesicular presynaptic protein. Whole coverslips were mapped and reconstructed with a confocal microscope (8×) and synaptophysin density appeared as a gray scale (from black = 0 to white = 255).

We then quantified synaptic networks made by neurons by analyzing synaptophysin pixel densities (Figure 6B). Values were expressed as a% of the total area, obtained for each PDL condition. Considering the 3 different PDL, less than 10% of the total area was covered by synaptophysin at 0 and 1 μg/ml (Figure 6B). PDL9 (Figure 6B; magenta curve) induced the lowest density of synaptophysin and was significantly different from GPDL9 (Figure 6B; cyan curve) and PDL6 (Figure 6B; yellow curve; two-way ANOVA and Tukey’s multiple comparisons test; respectively *p < 0.05 and **p < 0.01; *n* = 2–6 per group). Behavior of neurons grown on GPDL9 (Figure 6B; cyan curve) highly depended on its concentration. Indeed, the synaptophysin peak was reached at 5 μg/ml and then densities decreased with increasing concentrations (10, 20 and 40 μg/ml) of GPDL9. Interestingly, similar results were reported recently, with dendritic polyglycerol amine enhancing neuronal adhesion only at 10 μg/ml, but not at 1 μg/ml or 100 μg/ml (Clement et al., 2022). With increasing concentrations of PDL6 (Figure 5B; yellow curve), synaptophysin densities constantly increased, suggesting that a high concentration of PDL6 is required to properly support neuronal networks.

Higher magnifications (Figure 6C; 20 × ; synaptophysin puncta in red and GluR1 positive cortical neurons in green) highlighted differences in the development of cultures, depending on PDL conditions. Clusters were seen with PDL6 (Figure 6C, a; 20 μg/ml). GPDL9 (Figure 6C, b; 5 μg/ml) induced a higher density of GluR1 positive neurons and dense and expanded synaptic networks. Clustering was significantly decreased by 49 ± 15,7% with GPDL9 5 μg/ml, compared to PDL6 20 μg/ml (t-test; *p < 0.05; *n* = 3 cultures per condition). When adsorbed rather than grafted, PDL9 (Figure 6C, c; 5 μg/ml) led to a very poor presynaptic staining and only a few GluR1 positive neurons were observed. Increasing GPDL9 concentration induced a similar pattern (Figure 6C, d; 10 μg/ml).

These experiments allowed us to that validate GPDL9 is a suitable substrate for our cortical neurons, when used at 5 μg/ml. In the following parts of our study, we compared our “standard” coating condition, PDL6 at 20 μg/ml, to GPDL9 at 5 μg/ml, our new improved coating condition. Adsorbed PDL9 was excluded of our experiments from that point since it failed to properly support adhesion and maturation of cortical cultures.

### 4.5. The PDL substrate influences neurite growth and synaptic contacts

**FIGURE 7:**
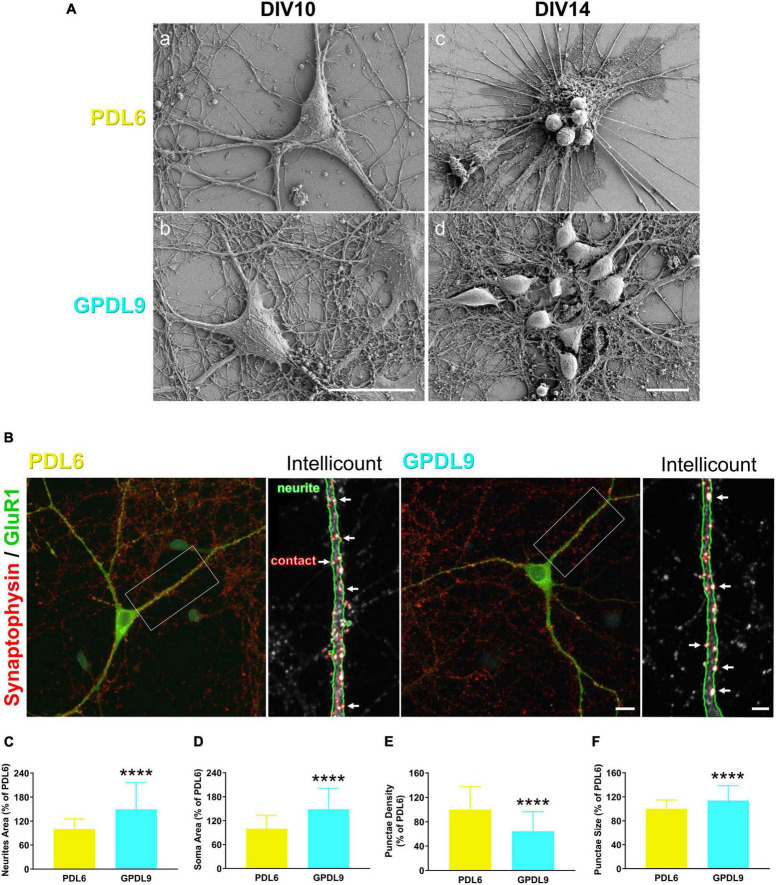
Effects of PDL type on neurite growth and synaptic contacts. **(A)** Scanning electron microscopy (SEM) images of individual neurons at DIV10 (a-b) or cell networks at DIV14 (c-d) plated on PDL6 (20 μg/ml; a,c) or GPDL9 (5 μg/ml; b,d; scale bars = 20 μm). **(B)** Confocal images of individual DIV14 neurons seeded on PDL6 (20 μg/ml; left panel) or GPDL9 (5 μg/ml; right panel), and immunostained with synaptophysin and GluR1 (green). Insets show analyzed images obtained with the Intellicount software, where neurites were delimited by green edges and synaptic contacts were identified by red puncta (white arrows). **(C–F)** Data were expressed as a% of the mean of PDL6. Quantification showed a significant difference of neurites area [**(C)** t-test; ****p < 0.0001], of soma area [**(D)** t-test; ****p < 0.0001], of puncta density [**(E)** t-test; ****p < 0.0001] and size of synaptophysin positive punctae [**(F)** t-test; ****p < 0.0001] between neurons seeded on PDL6 (20 μg/ml) or GPDL9 (5 μg/ml; scale bars 10 μm and 3 μm; *n* = 66–184 neurons).

Morphological observations of cortical neurons using scanning electron microscopy (SEM) at DIV10, confirmed a high level of network complexity on GPDL9 substrate, compared to PDL6 (Figure 7A; panel b *vs* panel a). Some clusters of rounded and small cells, from which thick bundles emerged, were seen at DIV14 on PDL6 (Figure 7A, c). On GPDL9, cell groups could also be seen at DIV14, but they showed healthy shapes and dense networks of neurites (Figure 7A, d).

DIV14 neurons were immunostained with synaptophysin (Figure 7B; red) and GluR1 (Figure 7B; green) and imaged at high magnification (100×) using a confocal microscope. Photomicrographs were analyzed using the Intellicount software (Fantuzzo et al., 2017). As seen on the insets, neurites were automatically delimited by green edges and synaptic contacts were identified by red puncta (Figure 7B; white arrows). Data were expressed as a% of the mean of PDL6. Quantification showed a significant increase of neurites (Figure 7C; t-test; ****p < 0.0001), and soma area (Figure 7D; t-test; ****p < 0.0001), as well as a significant decrease of puncta density (Figure 7E; t-test; ****p < 0.0001) and a significant increase of puncta size (Figure 7F; t-test; ****p < 0.0001) between neurons seeded on PDL6 (20 μg/ml) or GPDL9 (5 μg/ml; scale bars = 10 and 3 μm; *n* = 66–184). Thus, interestingly, the surface of neurons was affected by the type of substrate, and synaptic contacts were differently arranged in the two conditions of PDL.

### 4.6. The PDL substrate influences synaptic activity and neuron properties

**FIGURE 8:**
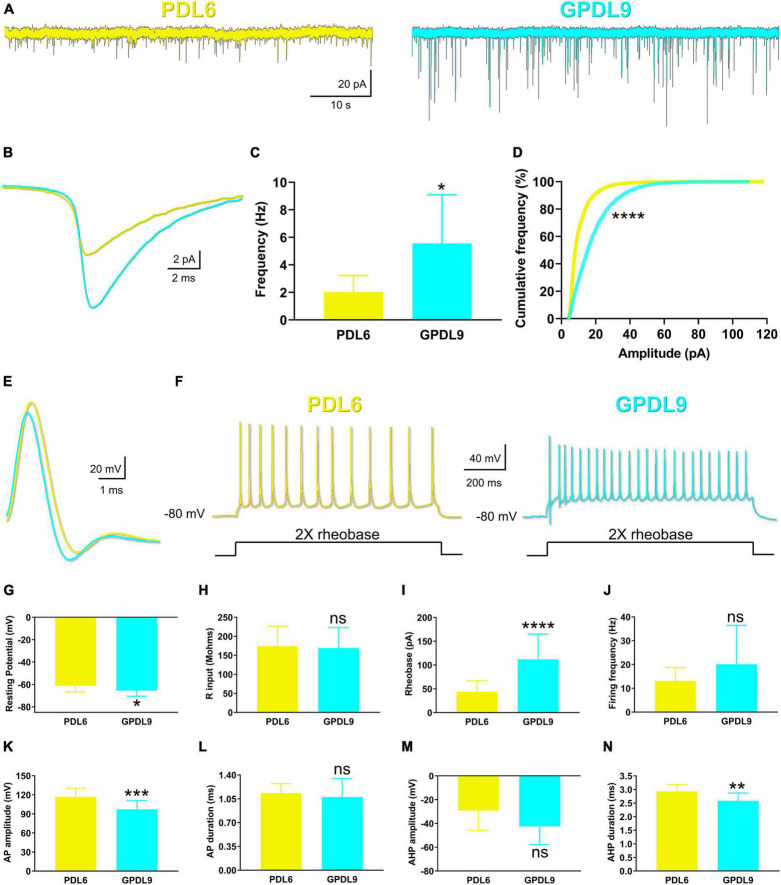
Effects of PDL type on synaptic activity and electrophysiological properties. **(A)** Example of miniature excitatory post-synaptic currents (mEPSCs) recorded from neurons seeded on PDL6 (20 μg/ml; left yellow trace) and GPDL9 (5 μg/ml; right cyan trace). **(B)** Average traces of mEPSCs recorded from neurons seeded on PDL6 (20 μg/ml; yellow trace) and GPDL9 (5 μg/ml; cyan trace). Frequency **(C)** and amplitude **(D)** of mEPSCs were significantly increased when neurons were seeded on GPDL9 compared to PDL6 (Welch’s test; *p < 0.05; ****p < 0.0001; *n* = 10–19). **(E)** Average traces of action potentials elicited near the rheobase in neurons seeded on PDL6 (yellow trace) or GPDL9 (cyan trace). **(F)** Response of recorded neurons seeded on PDL6 (left yellow trace) or GPDL9 (right cyan trace) to a 1 s depolarizing current pulse (twice the rheobase). Analysis of resting membrane potential [RMP **(G)**], input resistance [R input **(H)**], rheobase **(I)**, firing frequency **(J)**, action potential (AP) amplitude **(K)** and duration **(L)** and after hyperpolarization (AHP) amplitude **(M)** and duration **(N)** in neurons seeded on PDL6 (yellow bars) compared to GPDL9 (cyan bars; t-test; ns *p* > 0.05; *p < 0.05; **p < 0.01; ***p < 0.001; ****p < 0.0001; *n* = 10–24).

Since neurons seeded on PDL6 (20 μg/ml) or GPDL9 (5 μg/ml) showed significant morphological differences, we next investigated their functionality using patch clamp recordings. We were first interested in synaptic activity, and we recorded miniature excitatory post-synaptic currents (mEPSCs; Figures 8A, B; PDL6 = yellow traces, GPDL9 = cyan traces). Analysis of these synaptic events indicated a significant increase of frequency (Figure 8C; Welch’s test; *p < 0.05; *n* = 10–19) and amplitude (Figure 8D; Kolmogorov-Smirnov test; ****p < 0.0001; *n* = 10–19) for neurons seeded on GPDL9 (cyan bar and curve) compared to PDL6 (yellow bar and curve).

We then analyzed passive and active membrane properties of neurons (Figures 8E–N). Figure 8E shows average traces of action potential elicited near the rheobase, in neurons seeded on PDL6 (yellow trace) and GPDL9 (cyan trace) and Figure 8F presents the response of recorded neurons seeded on PDL6 (left yellow trace) and GPDL9 (right cyan trace) to a 1 s depolarizing current pulse (twice the rheobase).

Resting membrane potential (RMP; Figure 8G), rheobase (Figure 8I), action potential (AP) amplitude (Figure 8K) and after hyperpolarization duration (AHP; Figure 8N) were significantly different between the two groups (PDL6 = yellow bars, GPDL9 = cyan bars; t-test; *p < 0.05; **p < 0.01; ***p < 0.001; ****p < 0.0001; *n* = 10–24), while input resistance (R input; Figure 8H), firing frequency (Figure 8J), AP duration (Figure 8L) and AHP amplitude (Figure 8M) were not (t-test; ns *p* > 0.05; *n* = 10–24).

Our results indicated an increase spontaneous synaptic activity when neurons were seeded on GPDL9, and many electrophysiological parameters were significantly different between the two different coatings.

### 4.7. The PDL substrate influences neuronal network activity

**FIGURE 9:**
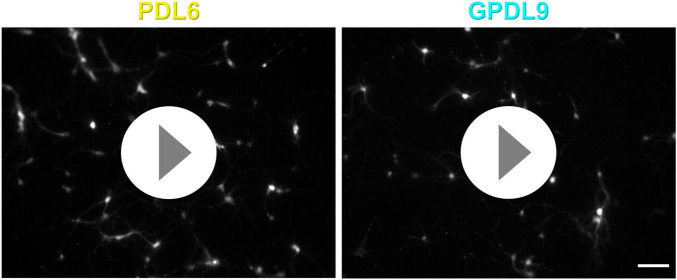
Videos 1 and 2: Calcium imaging recorded from cultures seeded on PDL6 (20 μg/ml left) or GPDL9 (5 μg/ml right), infected at DIV2 and imaged at DIV9. Scale bar = 100 μm. Videos are available online as Supplementary Files.

Electrophysiological recordings in individual neurons highlighted functional differences between the two different coating conditions. Then, to assess neuronal activity in many neurons simultaneously, we used calcium imaging. GCaMP6 is a family of ultrasensitive fluorescent calcium sensors developed for neuronal activity imaging (Chen et al., 2013). Cortical neuronal cultures were infected with a viral construct encoding GCaMP6 and then imaged (Figure 9). Videos 1 and 2 are available online as Supplementary Files.

**FIGURE 10:**
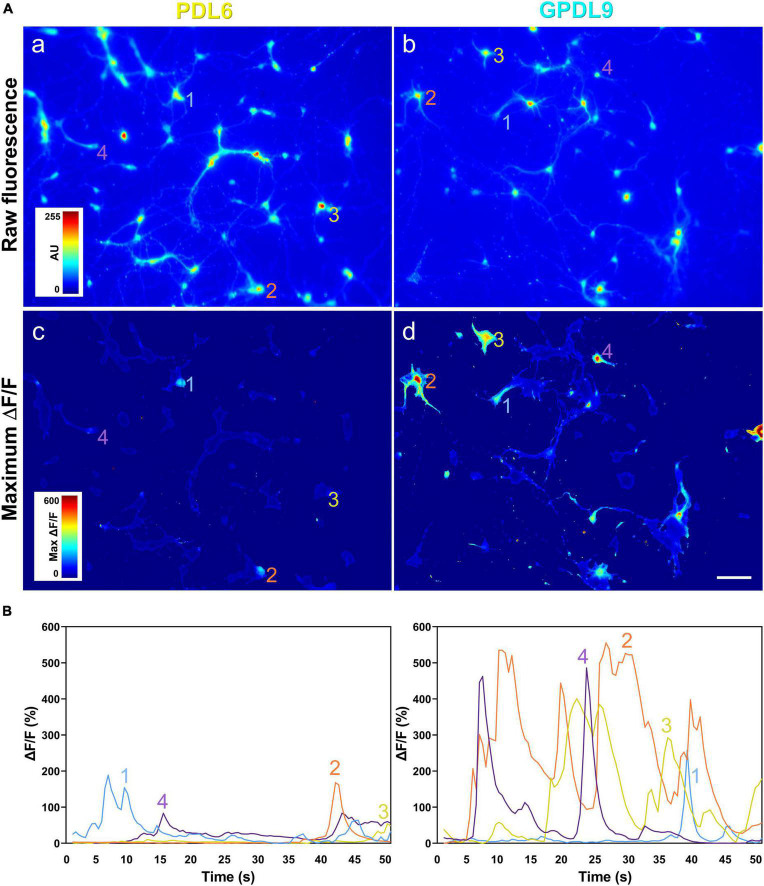
The PDL substrate influences neuronal network activity. **(A)** Raw fluorescence of neurons seeded on PDL6 [(a) left upper panel] or GPDL9 [(b) right upper panel], and image of maximum ΔF/F values over time, for neurons plated on PDL6 [(c) left lower panel] or GPDL9 [(d) right lower panel]. **(B)** Average values of the fluorescence within a region of interest of 11 × 11 pixels for 4 selected neurons seeded on PDL6 (left panel) or GPDL9 (right panel). Scale bar = 100 μm.

Changes in calcium indicator fluorescence, corresponding to calcium transients and spontaneous neuronal activity, were seen as flashes of different intensities, in both neurites and cell bodies. The spontaneous fluctuations of neuronal fluorescence were used the measure the network physiology under the two different coatings (Figures 10A, a,b). The maximum ΔF/F values were higher when neurons were seeded on GPDL9 (Figure 10A, d) compared to PDL6 (Figure 10A, c) and this was associated with an increase of the frequency and amplitude of calcium events (Figure 10B). Recordings of calcium activity *in vitro*, confirmed that networks of cortical neurons seeded on GPDL9 (Figure 10B, right panel), were functionally more active than networks of neurons seeded on PDL6 (Figure 10B, left panel).

## 5. Discussion

PDL is inexpensive, known as a well-characterized universal cell substrate, not degraded by cells, and stable in solution. Commercial PDL-coated coverslips are available, albeit expensive, and the coating procedure is not described by the manufacturer. Here, we proposed a new alternative, based on covalent binding of PDL on glass silanized coverslips (Figure 1). Our home-made protocol is advantageous as it is simple, economical, and the recipe can be controlled and adjusted on demand. Furthermore, batches of silanized coverslips can be prepared in advance, stored for a couple of weeks, and incubated with PDL when required.

We confirmed that the layer made with adsorbed PDL6 on glass coverslip was the main cause of reaggregation of primary cortical neurons, a few days after seeding (Figures 2C, D). This PDL-dependent phenomenon has been known for a long time (Lucas et al., 1986) and reported independently, more recently (Wang et al., 2018; Liu et al., 2019). Thus, Wang et al. (2018) showed aggregates and thick bundles when cerebellar granule neurons were seeded on PDL at low concentrations. Dissociated primary cortical neurons were also reported to adhere and then cluster on PDL by Liu et al. (2019). In these two papers, the PDL substrate was compared with new coating strategies, designed to improve adhesion but involved PDL dependent complex design (Liu et al., 2019), or PDL independent protocol (Wang et al., 2018).

Coomassie Brilliant Blue (CBB), a dye commonly used to stain proteins, was chosen here to easily observe the presence and the homogeneity of PDL coatings on coverslips (Figure 3), as recently reported for PLL-coated plastic on Petri dishes (Lin et al., 2019). Instead of using complex characterization techniques such as fluorescent-labeled PDL (Kim et al., 2011) or atomic force microscopy, we used the CBB staining to get both quantitative and qualitative results about the PDL layer (Figure 4). More specifically, PDL deposition conditions (time, pH and concentration) were optimized to obtain homogeneous PDL coatings on glass coverslips in a reproducible and replicable manner.

We next investigated neuronal behavior on various PDL substrates. When seeded on adsorbed PDL6 (1–40 μg/ml) or grafted GPDL9 (1–10 μg/ml), neurons adhered properly during an early phase (Figure 5; DIV4). Similar results were observed with rat primary cortical cultures at DIV3, with adsorbed PDL and covalently bound PDL on indium-tin oxide. Interestingly, clusters appeared after DIV7, only on adsorbed PDL (Kim et al., 2011). Here, we did not observe reaggregation with covalent binding either, at DIV10 (Figure 6C) and after. Hence, neuronal adhesion stability was shown to be efficiently enhanced with covalent binding of PDL on two different substrates.

To investigate neuronal maturation, we focused on synaptic markers including synaptophysin and GluR1 (Figures 7B, C). Area of GluR1 positive neurites was significantly increased in the GDPL9 group, suggesting a better contact of neurons with grafted PDL compared to adsorbed PDL. The number of synaptophysin positive inputs was similar in the two groups. However, because of the increased neurite area, the synaptic density was lower on neurons seeded on GDPL9. On another side, the mean size of synaptic puncta was significantly increased. In cortical neurons cultured from newborn mice, Soham et al., reported an increase of synapsin area, another synaptic protein, normalized with the area of MAP2, a dendritic marker, between DIV4 and DIV16 (Chanda et al., 2017). As illustrated in the review of Ziv and Garner (2004), precisely on their model for presynaptic assembly during neuronal maturation, a developing presynaptic bouton shows only a small reserve of synaptic vesicles, while a fully formed presynaptic bouton contains a large pool of vesicles. Interestingly, the synapse size is also associated with its stability, with new synapses being smaller compared to stable ones (Quinn et al., 2019). Our results strengthen the idea that covalent binding of PDL provides a more stable surface than adsorbed PDL, promoting neuronal maturation and assembly of mature synapses.

Covalent binding of PDL improves maturation of stem cell-derived neurons, by increasing the density of synapsin puncta (Kamande et al., 2019). However, in Kamande et al., morphological results were not confirmed by functional measures. Here, we have shown that covalent binding of PDL could influence neuronal activity through functional evidence, involving patch clamp recordings (Figures 8A–D), and calcium imaging (Videos 1 and 2, Figures 9, 10). Analysis of miniature synaptic excitatory events (mEPSCs) showed higher frequency and amplitude, and calcium imaging revealed a higher level of network activity, in neurons seeded on GPDL9 compared to neurons growing on PDL6. In cultured hippocampal neurons, a developmental increase of synaptic puncta size occurs in parallel with an increase of both frequency and amplitude of mEPSCs (Chanda et al., 2017), and Holler et al. (2021) showed a correlation between synapse size and synaptic transmission strength, on cortical slices of postnatal young mice. Thus, our results suggest that covalent binding of PDL influences not only morphological establishment of synaptic inputs, but also their functionality.

To go further, we analyzed intrinsic electrophysiological properties of primary cortical neurons. Neurons seeded on GPDL9 showed a significantly more hyperpolarized resting membrane potential (RMP; Figure 8G), compared to neurons seeded on PDL6. As shown in cultures of superior cervical ganglion neurons (Amendola et al., 2015), or in slices of developing rat visual cortex (Kasper et al., 1994), hyperpolarization of RMP is a universal hallmark of neuronal maturation. Insertion of ionic channels into the membrane is known to be responsible for maturation of many electrophysiological properties. RMP hyperpolarization has been associated with a developmental increase of Kir2 inwardly rectifying potassium channel family expression, in neocortical inhibitory interneurons (Goldberg et al., 2011). This type of potassium channel is also expressed on rat cortical pyramidal neurons and is responsible for their hyperpolarized resting potential (Day et al., 2005). We suggest that a differential maturation of Kir2 potassium channel family could explain PDL-dependent RMP values. Thus, a comparison of Kir2 expression between the two groups, PDL6 and GPDL9, and a specific pharmacological blockage of these channels could confirm this hypothesis. Neurons seeded on GPDL9 also showed a significant increase of rheobase (Figure 8I), a higher firing frequency (Figures 8F, J), and a significant decrease of AHP duration (Figure 8N) These features are also known to be related to mature neurons (McCormick and Prince, 1987; Larimer and Hasenstaub, 2020). Once more, neurons seeded on grafted PDL showed some characteristics of more mature cells, compared with neurons seeded on adsorbed PDL.

## 6. Conclusion

**FIGURE 11:**
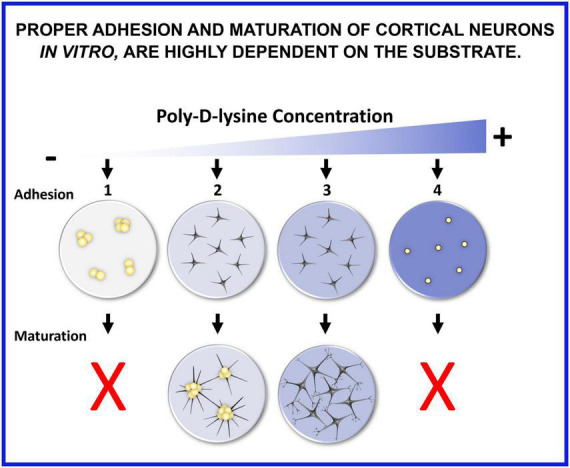
Proper adhesion and maturation of cortical neurons *in vitro* are highly dependent on the substrate. (1) If seeded on glass without any coating, neurons do not adhere, rapidly reaggregate, and their maturation is compromised. (2) When poly-D-lysine (PDL) concentration is too low or when PDL6 is adsorbed on glass coverslips, neurons can adhere, but slyly tend to cluster a few days after seeding. (3) When PDL coating is optimal and stable, especially with covalent binding, neurons adhere and develop expanded and functional synaptic networks. (4) A too high concentration of PDL is also detrimental because neurons do not attach, float, are small and rounded, and maturation cannot occur.

We have shown that proper adhesion and maturation of cortical neurons *in vitro* are highly dependent on the substrate (Figure 11). If seeded on glass without any coating, neurons do not adhere, rapidly reaggregate, and their maturation is compromised (1). When poly-D-lysine (PDL) concentration is too low or when PDL6 is adsorbed on glass coverslips, neurons can adhere, but slyly tend to cluster a few days after seeding (2). When PDL coating is optimal and stable, especially with covalent binding, neurons adhere and develop expanded and functional synaptic networks (3). A too high concentration of PDL is also detrimental because neurons do not attach, float, are small and rounded, and maturation cannot occur (4).

Covalent binding of PDL enhances neuronal adhesion stability, fixes the long-term reaggregation phenomenon, and improves neuronal maturation. Thus, choosing the appropriate coating for culturing neurons is crucial, and the same substrate should be kept between experiments to avoid evident bias. On this point, substrate differences should be considered for study comparisons, because they could sustain eventual data divergences seen in the literature. Comparing neuronal maturation and the establishment of stable synapses between different culture methods is not easy and could be biased because many other factors could influence these phenomena and their progress. Indeed, cultures made with various origins of neurons (species, brain region, age of embryos or newborn…), substrate, cell density, medium, *etc*…will not give identical functional parameters. For example, in hippocampal neurons cultured from newborn mice, Chanda et al. (2017) reported a developmental increase (between DIV4 and DIV16) of mEPSCs frequency from 0.25 Hz to 1 Hz. Here, in cortical neurons cultured from embryonic mice, the mean frequency of mEPSCs is 2 Hz for cells seeded on PDL6, and 5 Hz for cells seeded on GPDL9. However, the same conditions of culture could be compared with different substrates, and thus, only relative criteria, instead of absolute criteria, could be established, such as an increase of synaptic puncta area, a higher frequency and amplitude of mEPSCs, a more hyperpolarized RMP, an increase of rheobase, *etc*…as seen here and in developmental studies (McCormick and Prince, 1987; Kasper et al., 1994; Amendola et al., 2015; Chanda et al., 2017; Larimer and Hasenstaub, 2020).

As shown in the present study, covalent binding of PDL is suitable for very fragile and sensitive cells such as primary cortical neurons. Our method has been also successfully tested for primary hippocampal neurons (not shown here). Covalent binding of PLL has been used to coat glass slides and to enhance adhesion of cervical exfoliative cells, collected for cancer screening (Xing et al., 2012). Our simpler protocol could be easily adapted for this purpose, and could provide also a stable PL coating for many other applications, since PL is a universal substrate, based on the electrostatic interaction between the positively charged lysine groups of PL and the negative phospholipid bilayer of the plasma membrane (Nevo et al., 1955).

## Data availability statement

The raw data supporting the conclusions of this article will be made available by the authors, without undue reservation.

## Ethics statement

The animal study was reviewed and approved by the ethics committee on animal research of the Université de Montréal (CDEA; protocol numbers #21-058, #21-059, #22-057 and #22-058).

## Author contributions

AS: conceptualization, methodology, validation, formal analysis, investigation, data curation, writing—original draft, illustrations, supervision, project administration. BL: conceptualization, methodology, validation, investigation, supervision, project administration, writing—review and editing. DGB: methodology, validation, writing—review and editing. LL: methodology, writing—review and editing. LA: investigation, writing—review and editing. PP: formal analysis, writing—review and editing. AN: resources, writing—review and editing. ÉD: validation, writing—review and editing. TO-B: software, validation, writing—review and editing. MV: methodology, formal analysis, resources, writing—review and editing. GD: validation, resources, supervision, project administration, writing—review and editing. J-FB: validation, resources, supervision, project administration, funding acquisition, writing—review and editing. All authors contributed to the article and approved the submitted version.
